# Environmental Dimensionality Controls the Interaction of Phagocytes with the Pathogenic Fungi Aspergillus fumigatus and Candida albicans

**DOI:** 10.1371/journal.ppat.0030013

**Published:** 2007-02-02

**Authors:** Judith Behnsen, Priyanka Narang, Mike Hasenberg, Frank Gunzer, Ursula Bilitewski, Nina Klippel, Manfred Rohde, Matthias Brock, Axel A Brakhage, Matthias Gunzer

**Affiliations:** 1 Department of Molecular and Applied Microbiology, Leibniz Institute for Natural Product Research and Infection Biology, Hans Knöll Institute, Jena, Germany; 2 Helmholtz Centre for Infection Research, Braunschweig, Germany; 3 Department of Physics, The German University of Cairo, New Cairo City, Egypt; Johns Hopkins, United States of America

## Abstract

The fungal pathogens Aspergillus fumigatus and Candida albicans are major health threats for immune-compromised patients. Normally, macrophages and neutrophil granulocytes phagocytose inhaled *Aspergillus* conidia in the two-dimensional (2-D) environment of the alveolar lumen or *Candida* growing in tissue microabscesses, which are composed of a three-dimensional (3-D) extracellular matrix. However, neither the cellular dynamics, the per-cell efficiency, the outcome of this interaction, nor the environmental impact on this process are known. Live imaging shows that the interaction of phagocytes with *Aspergillus* or *Candida* in 2-D liquid cultures or 3-D collagen environments is a dynamic process that includes phagocytosis, dragging, or the mere touching of fungal elements. Neutrophils and alveolar macrophages efficiently phagocytosed or dragged *Aspergillus* conidia in 2-D, while in 3-D their function was severely impaired. The reverse was found for phagocytosis of *Candida.* The phagocytosis rate was very low in 2-D, while in 3-D most neutrophils internalized multiple yeasts. In competitive assays, neutrophils primarily incorporated *Aspergillus* conidia in 2-D and *Candida* yeasts in 3-D despite frequent touching of the other pathogen. Thus, phagocytes show activity best in the environment where a pathogen is naturally encountered. This could explain why “delocalized” *Aspergillus* infections such as hematogeneous spread are almost uncontrollable diseases, even in immunocompetent individuals.

## Author Summary

Aspergillus fumigatus and Candida albicans are the most common of all human pathogenic fungal germs. Normally, inhaled *Aspergillus* spores are destroyed by alveolar macrophages and polymorphonuclear neutrophils (PMNs), both of which are lung phagocytes, i.e., cells that kill inhaled microbes by ingestion. In contrast, C. albicans is a normal constituent of the human gut flora that is controlled by tissue-resident PMNs. If immune control is lost, both fungi grow into the surrounding tissue and cause life-threatening infections. To investigate how phagocytes function in the disparate environments of lung air sacs (lacking a definite matrix-composition [two-dimensional (2-D)]) or mucosal tissues (providing a three-dimensional [3-D] space), the authors mimicked 2-D and 3-D environments and analyzed the process of ingestion, called phagocytosis, by PMNs and other phagocytes. Phagocytosis was a dynamic cellular process where distinct cells showed vastly different behavior. The environmental setup (2-D versus 3-D) had a profound impact on the cell's ability to phagocytose. *Aspergillus* conidia were much better ingested in 2-D systems, while *Candida* yeasts were only ingested in 3-D systems, even if the other pathogen was present. This was true for different 2-D and 3-D systems and for both cells of mice and humans. Besides providing a comprehensive analysis of the cellular movements underlying phagocytosis, the results also suggest an evolution of phagocytes to optimally recognize fungal pathogens in the environment of natural infection.

## Introduction

The frequency of invasive mycoses due to opportunistic fungal pathogens has increased significantly over the past decades. These infections are associated with high morbidity and mortality. They directly correlate with increasing patient populations that are at risk for developing invasive fungal infections. These populations include individuals undergoing solid-organ transplantation, bone marrow transplantation, and major surgery, as well as those with AIDS, neoplastic disease, immunosuppressive therapy, advanced age, and premature birth [1–3]. In this context, Aspergillus fumigatus can be regarded as the primary mold pathogen.

Conidia of A. fumigatus are constantly inhaled. However, in immunocompetent individuals, mucociliary clearance and phagocytic defense prevent the disease. Alveolar macrophages (AMs) are the major phagocytes of lung alveoli. They, along with polymorphonuclear neutrophils (PMNs), which are recruited during inflammation, are responsible for phagocytosis of A. fumigatus [1,4–6]. AMs kill conidia by producing reactive oxygen species [7]. The conidia that escape from the AMs germinate, but are attacked by PMNs that adhere to the surface of the hyphae and kill them by secretion of reactive oxygen species and degranulation [8–14]. In addition, PMNs are also able to kill resting or swollen conidia [1,6]. Furthermore, A. fumigatus antigens induce the activation and maturation of dendritic cells (DCs) [8]. A dysfunctional immune system, however, provides an opportunity for conidia to germinate and invade lung tissue [6,9].

Candida albicans is a normal component of mucosal flora, which in immunocompromised individuals can transform itself into an invasive pathogen [10]. The primary defense of the mucosal flora is formed by PMNs [11], which are rapidly recruited to the sites of infections [12] and phagocytose the fungus in the tissue environment of microabscesses [13].

The process of A. fumigatus phagocytosis has been analyzed in detail using AMs from mice [7] or humans [14,15], macrophage cell lines [15,16], or DCs from the mouse [17]. In addition, the interaction of PMNs with hyphae in the presence of platelets has been studied [18]. However, none of these studies enabled the direct observation of the process itself by live cell imaging, since the available techniques did not allow the direct distinction between phagocyte and fungal components. Also, live cell imaging of phagocytosis of *Candida* has not been performed to our knowledge. Cell motility, however, is an essential requirement for the function of phagocytes. Defects in the cellular dynamics as a basis for defects in function are well known. One such example is that inhibition of the cytoskeleton of DCs [17] or AMs [15] severely inhibits the ability of these cells to phagocytose *Aspergillus* conidia. And genetic defects in actin polymerization are associated with increased susceptibility to infections [19].

Furthermore, it is generally assumed that physical contact between a phagocyte and a conidium inevitably leads to phagocytosis of the latter. Thus, it is unclear why previous studies addressing phagocytosis of A. fumigatus in vitro only found 50% of the phagocytes carrying conidia, while 50% of cells did not [7]. In addition, although PMNs are not very efficient at phagocytosing A. fumigatus in vitro, patients suffering from neutropenia are at a higher risk for developing invasive aspergilloses. Thus, mechanisms distinct from phagocytosis must exist, which allow PMNs to control fungal growth in the lung.

Finally, until now studies on phagocytosis have ignored the location in the body at which this process occurs. One of the major features of in vivo phagocytosis is its almost ubiquitous three-dimensionality, in other words, the spatial arrangement of extracellular matrix proteins that acts as a scaffold for the attachment and movement of cells [20]. While conventional experimental setups, where cells are cultured in dishes covered with a liquid medium, constitute a two-dimensional (2-D) system that does not allow migration in space, we mimic more physiological conditions by providing a three-dimensional (3-D) collagen matrix as a substratum for cell migration and cell–cell communication [21,22]. We have previously used this system to demonstrate strong differences in the interaction of T cells with antigen presenting cells in 3-D versus 2-D environments [23]. Subsequently, the results have been confirmed by us [24] and others [25–27] in true lymphatic tissue.

However, in vivo 2-D movements can also be observed, and phagocytosis of pathogens at the surface of a lung alveolus [28] is a prominent example of this. The inner part of an alveolus is a surface composed of type-I epithelial cells, which is covered by a water-based hypophase and a thin film of surfactant produced by type-II epithelial cells. Hypophase and surfactant form the surface-lining layer (SLL) [29]. The SLL is not a solid, migration-supporting scaffold but rather is thin (in the range of 50 nm [30]) and devoid of prominent internal structures [30–32]. Inhaled particles such as A. fumigatus conidia are drawn beneath the SLL, pressed to the epithelial surface by surface tension, and then are phagocytosed [30,33]. Normal cellular motility is essential for this process, as impaired motility in the thick mucus isolated from the lungs of individuals with cystic fibrosis is associated with decreased phagocytic activity of PMNs [34]. In contrast, alveolar DCs, the major antigen-presenting cells of the lung, must be able to phagocytose conidia from the interstitial tissue between alveoli, which is composed of collagenous fibers [35], and thereafter migrate from the lung into draining lymph nodes to present pathogenic antigens to T cells [17].

Thus, this study was performed to obtain a dynamic picture of the phagocytosis of A. fumigatus and C. albicans by the main immune-effector cells. The goal was to directly compare the phagocytic efficiency on a per cell basis to elucidate the fate of individual fungal elements and finally study the role of environmental cues on the observed mechanisms.

## Results

### Generation of an A. fumigatus Strain Expressing DsRed2

**Figure 1:**
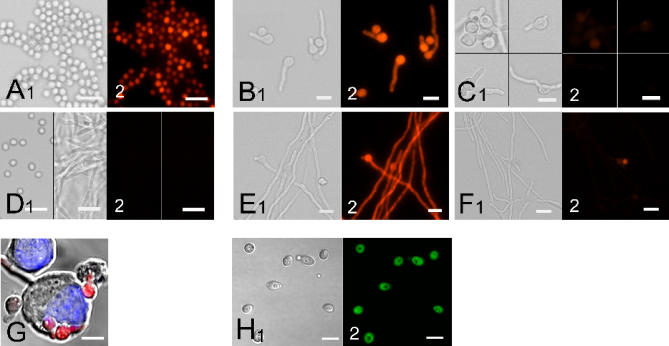
Fluorescence of A. fumigatus and C. albicans Strains Under Different Growth Conditions A transgenic strain of A. fumigatus expressing *DsRed2* under the control of the isocitrate lyase promoter (*acuD*p) was generated. To investigate the intensity of fluorescence as well as the dependency of the transgene expression on the carbon source, A. fumigatus was cultivated in AMM containing either glucose (A, C, and F) or ethanol (B, D, and E) as the sole carbon source. Different developmental stages were analyzed by light (panels 1) and fluorescence microscopy (panels 2). Resting conidia were derived from sporulating cultures on AMM agar plates containing glucose (A). Conidia were incubated in AMM for 7 h and 16 h to yield germlings (B and E) and hyphae (C and D), respectively. Identically treated wild-type conidia/hyphae are shown in (D). Fluorescence was observed irrespective of the carbon source in resting conidia. By contrast, germlings and hyphae only showed fluorescence when grown on ethanol. To test whether phagocytosis could activate the *acuD* promoter, we observed conidia germinating within macrophages 6 h after phagocytosis by J774 macrophages (G). An overlay is shown of the transmission light image, a DAPI stain for the nucleus (blue) and the red fluorescence of germinating conidia (G). To obtain green fluorescent C. albicans yeast cells, a number of cells were stained with the green cytoplasmic dye CFSE. A transmission light image (H1) and a fluorescence image (H2) of a fresh preparation of CFSE^+^ C. albicans yeast cells are shown. Bar = 5 μm.

The labeling of C. albicans with carboxyfluorescein-succinimidyl-ester (CFSE) was a reliable procedure and yielded bright and stable fluorescent cells useful for live cell imaging (Figure 1H). However, the same protocol did not work sufficiently for A. fumigatus. Thus, we generated a strain of A. fumigatus with bright and stable endogenous fluorescence by producing the red fluorescent protein DsRed2 using the *acuD* promoter, which gave conidia with strong red fluorescence, and which is differentially regulated in hyphae of A. fumigatus [36].

The 5′ sequence, including the ATG start codon and codons encoding some N-terminal amino acids of the isocitrate lyase gene *acuD,* was fused in frame with the *DsRed2* gene. All strains with an integration of the *acuD*p*-DsRed2* plasmid exhibited detectable fluorescence well. One of the strains, designated AcuD-DsRed2–9, was selected for further investigations. Southern analysis revealed that the strain contained two copies of the plasmid integrated at ectopic sites into the genome (unpublished data).

The expression of the *DsRed2* gene fusions was monitored by growing the transformants in *Aspergillus* minimal medium (AMM) for 7 h (germlings), or 16 h (hyphae), and on AMM agar plates for 5 d with different carbon sources at 37 °C. Different developmental stages of *A. fumigatus,* i.e., conidia, germlings, and hyphae, were studied. Conidia of AcuD-DsRed2–9 were derived from sporulating cultures on AMM agar plates and displayed strong fluorescence of stably deposited DsRed2 irrespective of the carbon source used (Figure 1A shows glucose, unpublished data with ethanol as carbon source). In contrast to conidia, fluorescence of germlings and hyphae was dependent on the carbon source. Strong fluorescence was observed in germlings and hyphae during cultivation on ethanol (Figure 1B and 1E). Cultivation of the fungus on glucose only led to faint, residual fluorescence in germlings (Figure 1C) whereas hyphae showed no fluorescence (Figure 1F), as did conidia or hyphae of the wild-type controls (Figure 1D). The observed slight fluorescence of germlings was possibly due to diffusion of the stable DsRed2 from red conidia into germlings. The carbon source-dependent expression pattern of *DsRed2* observed here, verified that the isocitrate lyase promoter is exclusively active during growth conditions that require the glyoxylate cycle, i.e., with ethanol or C2-generating carbon sources. Furthermore, bright fluorescence was observed when the conidia germinated in macrophages (Figure 1G), suggesting that isocitrate lyase plays a significant role while growing in macrophages.

### Live Imaging of DsRed2-Labeled Conidia Interacting with the Major Phagocytes Demonstrates Strong Influence of the Spatial Dimension of the Environment on Phagocytosis

**Figure 2:**
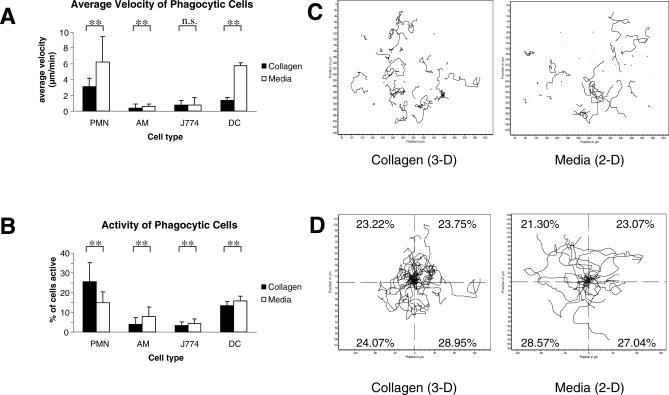
The Migration of Phagocytic Cells in 2-D and 3-D Environments Unstimulated cells (except J774 cells, which were stimulated overnight with 2.5 U/ml interferon-γ before use) were embedded along with A. fumigatus conidia within 3-D collagen matrices or within a 2-D liquid based chamber, and cell movements were recorded for 2 h by time-lapse video microscopy. The migration of 40 to 60 individual cells for each cell type was quantified by computer-assisted, single-cell tracking. (A and B) The average velocity (in μm/min) (A) and activity (percentage of migrating cells at a given time point) (B) were calculated from the tracks and are shown here as a comparison between different cell types. Data represent the average of three independent experiments. (C) Representative tracks of 40 PMNs were analyzed in a 3-D collagen matrix or in a 2-D media-based system. (D) The same cell tracks as depicted in (C) have been redrawn to artificially start at the center of the graph, and each individual step in each track was analyzed for its length and orientation within the four quadrants. This made it possible to measure the distance of all steps that were made into each quadrant. The numbers indicate the percentage of the total distance covered by all tracked cells in each of the four quadrants in 3-D and 2-D systems, showing almost random migration (perfect random migration would result in 25% distance in each quadrant). **, *p* < 0.01; n.s., not significant. Error bars represent standard deviation (SD).

Analysis of living cells by time-lapse microscopy and single-cell tracking showed that despite dynamic lamellipodia at the cell perimeter, neither AMs nor J774 cells migrated with a high velocity in either 2-D or 3-D environments (Figure 2A). This is in accordance with our earlier data on macrophage migration [21]. PMNs and DCs, in contrast, efficiently migrated in both 2-D and 3-D environments; however, migration in the 3-D environment was somewhat slower than that in the 2-D environment. The percentage of cells in a tracked population migrating at any given time (activity) was significantly higher for AMs, J774 cells, and DCs in 2-D versus 3-D systems. Conversely, a significantly higher number of PMNs was found to be more mobile in 3-D systems than in 2-D systems (Figure 2B). By analyzing tracks of single cells, we found that the migration of immune cells was almost perfectly random in all directions (Figure 2C and 2D).

**Figure 3:**
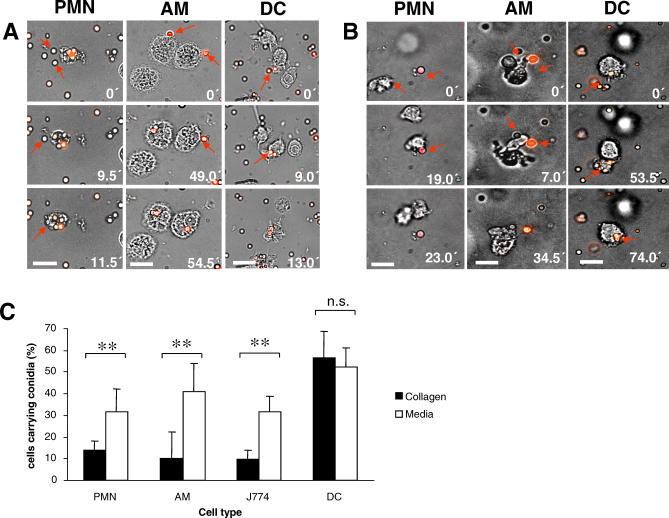
Phagocytosis of A. fumigatus Conidia by Different Phagocytes Is Dependent on the Dimensionality of the Environment (A) A pure fraction of immune cells and conidia was incorporated in a 2-D liquid-based system and subjected to time-lapse video microscopy. Panels show ingestion of conidia (marked by red arrows) by PMNs, AMs, and DCs. All cells were imaged over a period of 3 h. (B) Image sequences from PMNs, AMs, and DCs shown with A. fumigatus conidia in a 3-D collagen matrix. The left column time series displays the interaction of a red A. fumigatus conidium (indicated by red arrow) with a PMN and the latter's inability to ingest or drag the conidium despite contact. The middle column shows the unsuccessful interaction of an AM over a period of 2 h. The right column shows the efficient uptake of four conidia by a DC during a 3-h period. Bar = 25 μm. (C) A histogram quantitatively showing the influence of the environment (3-D, solid bar; 2-D, white bar) on the phagocytic ability of different cell types. The number of cells that had internalized conidia was counted 30 min after the start of imaging. The data represent the average of the percentage of cells internalizing conidia from three independent experiments, representing a total of 433 DCs in collagen, 356 DCs in media, 77 AMs in collagen, 229 AMs in media, 706 PMNs in collagen, 458 PMNs in media, 367 J774 cells in collagen, and 212 J774 cells in media. *, *p* < 0.05; **, *p* < 0.01; n.s., not significant. Error bars indicate SD.

The interaction of phagocytes with conidia of A. fumigatus was a highly dynamic process. In 2-D environments, actively migrating PMNs and DCs could be observed touching and phagocytosing multiple conidia within 1.5 h of observation (Figure 3A; Videos S1 and S2). Phagocytosis by AMs was less dynamic. Here, cells produced slight membrane protrusions towards nearby conidia before ingesting them (Figure 3A; Video S3). The phagocytosis of the macrophage cell line J774 was morphologically very similar to that of AMs. Here, we also observed that a single successful phagocytosis event could prime cells for more rapid additional phagocytosis events (Video S4).

However, when transferred into a 3-D tissue-based environment, PMNs, AMs, and J774 cells were impaired in their ability to phagocytose conidia. This was observed despite frequent touching that could even lead to a slight displacement of individual conidia (Figure 3B; Video S5). DCs, in contrast, were equally efficient at ingesting conidia in 3-D as compared with the 2-D environment (Figure 3B; Video S6). A quantification of these phenomena showed that DCs were the most efficient cells for the phagocytosis of conidia, both in 2-D and 3-D environments, while all other phagocytes efficiently phagocytosed in a 2-D environment but were severely limited in their capacity for phagocytosis in 3-D (Figure 3C).

### Dragging as a Major Mechanism for the Interaction of PMNs and DCs with Conidia of *Aspergillus*

**Figure 4:**
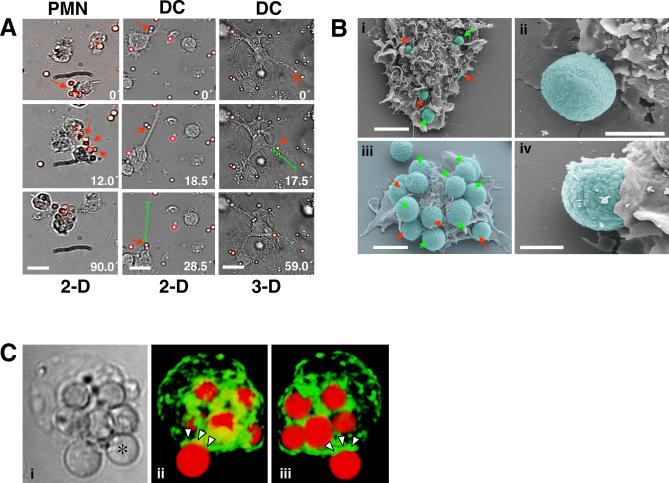
Dragging Is a Major Interaction Type of DC or PMN with Conidia of A. fumigatus (A) Time series from videos of A. fumigatus conidia and PMNs and DCs in 2-D and in 3-D systems show dragging as an alternative way of interaction between phagocytes and A. fumigatus conidia. The conidia are dragged by the PMNs in the form of a cluster. DCs are able to drag conidia over several micrometers (shown in green). Bar = 25 μm. (B) Electron microscope images of DCs (i) and (ii) and PMN (iii) and (iv) are shown interacting with conidia, which were pseudocolored blue to enhance contrast. Dragging and ongoing phagocytosis as seen in time-lapse imaging is evident in the form of several conidia attached to a single DC or PMN (shown here in green arrows), as well as in the process of being incorporated into (indicated by red arrows) a single DC or PMN in the high resolution electron microscopy image (Bar = 5 μm). (ii) shows an attached conidium to a DC, and (iv) shows a conidium being phagocytosed by a PMN (Bar = 2μm). (C) Attached and internalized conidia associated with the same PMN. PMNs were fixed and permeabilized during interaction with DsRed conidia. Subsequently, cells were stained with Alexa 488 labeled phalloidin. Two-color confocal microscopy was used to obtain a z stack covering the entire cell thickness. Shown is the transmission light image (i) as well as two views of a voxel rendering of composite red (fungus) green (actin) fluorescence images. (ii) corresponds to the same view as (i) and (iii) corresponds to a 180-degree rotation in the plane of the paper showing that five conidia are entirely covered by actin cytoskeleton pockets, while the sixth conidium is in the process of being phagocytosed as depicted by actin protrusions (arrowheads). These protrusions correspond to those depicted in (B) (iv). The conidium marked with an asterisk is not visible in the fluorescence image due to the loss of fluorescence. Also refer to Video S9.

A frequent characteristic of phagocyte–conidium interaction was the dragging of conidia over long distances without obvious phagocytosis. This was especially pronounced in PMNs in 2-D environments and, to a lesser extent, also with DCs (Figure 4A; Videos S7 and S8). The dragging of multiple conidia by a single PMN often led to the formation of large aggregates of nonphagocytosed conidia in the center surrounded by several PMNs on the periphery (Figure 4A; Video S7). High-resolution electron microscopy showed that dragging cells could generate surface extensions that were reminiscent of phagocytic cups to individual conidia, while other conidia on the same cell were merely attached to the cell surface without the induction of membrane protrusions (Figure 4B). Three dimensional reconstructions of confocal z stacks of PMNs incubated with conidia for 1 h clearly showed conidia completely internalized, and those conidia simply attached to the surface of a cell (Figure 4C; Video S9). Due to their inherently low mobility, neither AMs nor J774 cells dragged conidia.

### The Environment Directly Influences Touching, Dragging, and Phagocytosis of Individual Cells

**Figure 5:**
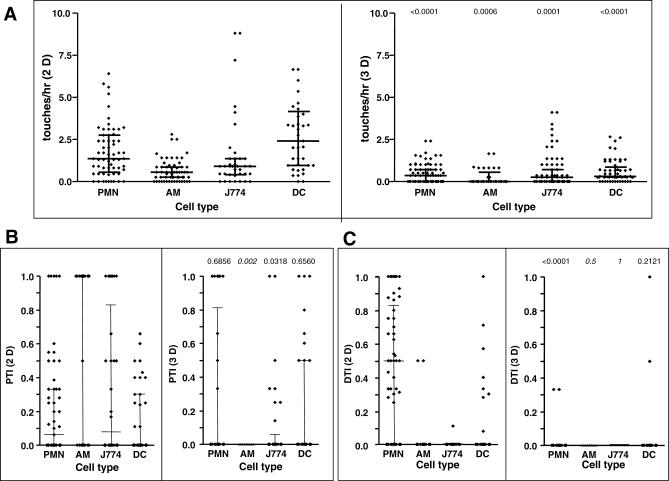
The Physical Interaction of A. fumigatus Conidia and Several Phagocytes Is Strongly Influenced by the Dimensionality of the Environment (A) Column scatter plots showing the number of contacts between phagocytes and conidia per hour in 2-D and 3-D environments. Each dot represents all observable contacts of an individual cell with conidia within a given video. As observation times for single cells vary greatly due to movements in and out of focus, values have been normalized on the average number of contacts per hour. Individual conidia touching cells as detected in (A) were subsequently followed for their fates. This could be simple release, dragging, or phagocytosis. Dots in (B) show the rate of conidia that have been successfully phagocytosed relative to the number of conidia that have been touched by a cell (PTI). Each dot represents the value for one single cell. All cell types have been investigated for their PTI in 2-D and in 3-D environments. (C) DTI values for the four cell types in 2-D and in 3-D environments. Evaluation of the DTI is analogous to the evaluation of the PTI with dragging as the physical interaction scored. Significance values (indicated over 3-D figures) have been obtained using the Mann-Whitney nonparametric *U*-test except where written in italics. Here the Mann-Whitney *U*-test was not applicable, and *p*-values were derived from the Wilcoxon rank sum test. Alternately, the Mann-Whitney *U*-test was performed after assigning arbitrary small values to the 0 entries. This yielded results similar to those of the Wilcoxon rank sum test (*p-*values not shown). The horizontal bars in each column scatter plot indicate median. The vertical lines represent interquartile ranges.

To study the frequency of conidium–phagocyte encounters, we analyzed the physical interaction of single cells with individual conidia in time-lapse video sequences (Figure 5). These analyses showed that touching rates between all types of phagocytes and conidia were higher in 2-D than in 3-D environments, reaching mean values between one to three contacts per cell per hour (Figure 5A). Individual DCs were found touching as many as ten conidia in 1 h in 2-D systems (Figure 5A). The percentage of cells touching conidia was consistently higher in 2-D environments, although, with the exception of AMs, these differences were not large enough to reach the level of significance (Table 1).

**Table 1:**
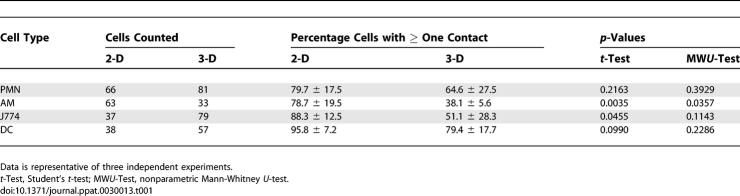
Percentage of All Cells with at Least One Contact with A. fumigatus Conidia in 2-D and 3-D

The continuous filming of cells interacting with conidia also allowed the analysis of the fate of touched conidia. Thus we calculated the rate of touches (phagocytosis touching index [PTI]) that finally ended in successful phagocytosis as shown in Videos S5–S8. These analyses showed, that for J774 cells, the PTI was lower in 3-D compared with that in 2-D environments. For AMs, the PTI in the 3-D environment was zero, meaning that none of the observed touches between AMs and conidia led to phagocytosis. For PMNs, fewer cells with an intermediate PTI were found in 3-D environments as compared with those in 2-D environments. At the same time, cells with a PTI of one remained equally as frequent. These differences, however, were not significant. Also, the PTI values of DCs did not change significantly with the environment (Figure 5B).

In the same way, we also analyzed the rate of touches that were followed by dragging the conidia rather than by phagocytosis (dragging touching index [DTI]). Here, the efficient dragging of conidia by PMNs in the 2-D environment was almost completely lost in the 3-D environment (Figure 5C). These data indicated that after transfer from a 2-D into a 3-D environment, physical contact formation between PMNs, AMs, and J774 cells with conidia was impaired, leading to strong defects in dragging and phagocytosis by these cells.

### Phagocytosis of C. albicans Is Optimal in 3-D Environments

**Figure 6:**
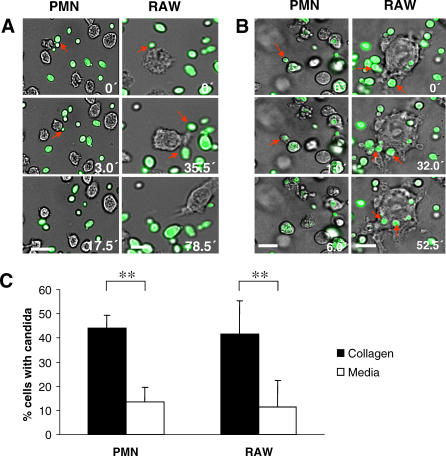
Phagocytosis of C. albicans by PMN and RAW Cells Is Enhanced in 3-D Environments (A) Image sequences from videos with PMN and RAW cells interacting with C. albicans in a 2-D system. Bar = 25 μm. The images show several contacts between the cells and the fungus (red arrows) without any ingestion or dragging. (B) Time series showing efficient uptake of C. albicans (red arrows) by PMN and RAW macrophages. Image sequences from videos with PMN and RAW cells interacting with C. albicans in a 3-D collagen matrix. Bar = 25 μm. Interactions were observed over a period of 3 h. (C) Quantitative representation of the effect of the environment on the phagocytosis of C. albicans by PMN and RAW cells in 2-D and in 3-D environments. The number of cells that had internalized C. albicans cells was counted 30 min after the start of imaging. The data represent the average percentage of cells internalizing yeasts from three independent experiments, representing a total of 241 PMNs in collagen, 187 in media, and 141 RAW macrophages in collagen, 97 in media. **, *p* < 0.01. Error bars represent SD.

AMs are exclusively located and function in the lungs, whereas PMNs can exert their activity in all tissues. Thus, we asked whether the defect in productive interaction with A. fumigatus conidia in 3-D environments was a general inability of PMNs to function in 3-D environments, or whether the type of pathogen determined cellular activity. Therefore, we measured the phagocytosis of the tissue-invading fungus C. albicans by PMNs and the macrophage cell line RAW 264.7, which is derived from peritoneal (i.e., tissue-associated) macrophages [37]. Live cell imaging showed intense contacts of both PMNs and RAW cells with yeast cells of C. albicans in 2-D (Figure 6A; Videos S10 and S11) as well as in 3-D environments (Figure 6B; Videos S12 and S13). Nevertheless, only a few PMNs or RAW cells were found carrying C. albicans intracellularly in the 2-D environment, while the number of cells carrying single or multiple yeast cells in 3-D systems was almost four times higher when compared to the 2-D systems (Figure 6C). Videos also showed the tendency of both PMNs (Video S12) and RAW cells (Video S13) to catch C. albicans cells even from great distances, while frequent contacts between phagocytes and C. albicans in 2-D environments led to no apparent physical association or a directional change of morphology of the cells.

**Figure 7:**
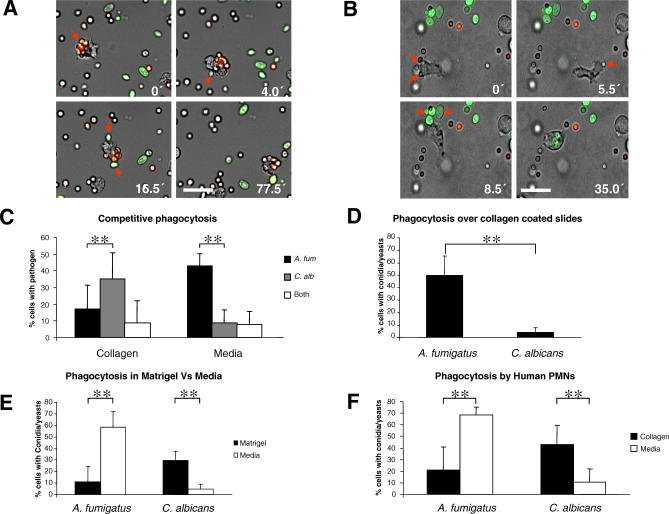
Phagocytosis of A. fumigatus and C. albicans Is Dependent on the Dimensionality of the Environment PMNs were incorporated along with A. fumigatus conidia and C. albicans yeast cells in the same system (2-D or 3-D), and the interactions were observed over a period of 3 h. (A) A PMN selectively taking up and dragging A. fumigatus conidia despite several contacts with C. albicans yeasts in a 2-D liquid system. Bar = 25μm. (B) Time series of a PMN selectively taking up three C. albicans cells despite touching at least four A. fumigatus conidia in 3-D (red arrows). Bar = 25 μm. (C) Quantitative representation of cells with internalized A. fumigatus conidia or C. albicans yeast cells or both in 2-D or 3-D systems. Data represent the average percentage of cells carrying conidia and/or yeasts from three independent experiments, representing 140 PMNs in collagen and 217 PMNs in media with error bars denoting SD. **, *p* < 0.01. (D) Quantitative comparison of the percentage of PMNs internalizing conidia or yeasts over collagen coated slides. The graph represents data from three independent experiments. Error bars denote SD. **, *p* < 0.001. (E) Average percentage of PMNs internalizing A. fumigatus or C. albicans in Matrigel (solid bar) and in media (open bar). Data represent the average from three independent experiments. Error bars indicate SD. **, *p* < 0.001 for both pathogens in 2-D and in 3-D environments. (F) Average percentage of PMNs derived from peripheral human blood with internalized conidia or yeasts in 2-D or 3-D systems. Data represent the average of cells taken from three healthy blood donors, each analyzed for all four conditions. Error bars indicate SD. **, *p* < 0.001.

Due to the previous observation that one completed phagocytosis event could increase the frequency of subsequent events (Video S4), we reasoned that successful interaction with one pathogen might enable PMNs to phagocytose the other pathogen en route, even within the “wrong” environment. To test this assumption, we set up competitive phagocytosis assays, where PMNs were allowed to interact with a mixture of A. fumigatus conidia and C. albicans yeast cells in both 2-D and 3-D environments. Again, imaging showed intensive contacts of PMNs with both fungal elements in both environments. However, in 2-D environments, PMNs selectively chose A. fumigatus conidia in a manner indistinguishable from the situation with conidia alone (Figure 7A; Video S14), while in 3-D environments very efficient phagocytosis of C. albicans occurred despite clearly detectable contacts to nearby A. fumigatus conidia by the same PMN (Figure 7B; Video S15). A quantification of these results showed that even in competitive phagocytosis assays, individual PMNs could phagocytose one pathogen very efficiently while simultaneously ignoring the other (Figure 7C).

It was possible that the contact with collagen fibers, rather than the dimensionality, was responsible for the observed differences in phagocytosis. To test this assumption, we analyzed the phagocytosis of either A. fumigatus or C. albicans by PMNs on slides coated with collagen. Here, cells were in intimate contact with collagen fibers, but were not embedded three-dimensionally within the matrices. Interestingly, on collagen-coated slides, PMNs were as efficient in interacting with A. fumigatus conidia as observed before in our conventional (plastic dish–based) 2-D system. Accordingly, C. albicans yeast cells could not be phagocytosed by PMNs on collagen-coated slides (Figure 7D). This suggested that it was indeed 3-D embedding within collagen that caused the observed differences in interaction/phagocytosis. The next question was whether this was a specific function of 3-D gels based on type-I collagen only. To test this, we measured interaction of PMNs with A. fumigatus conidia or C. albicans yeast cells in 3-D matrices composed of Matrigel (a basement membrane entirely devoid of type-I collagen). Its main constituents are laminin and type-IV collagen. Confirming our previous assumptions, the interaction of PMNs with both fungal pathogens within 3-D matrices composed of Matrigel was indistinguishable from what had been observed before within 3-D collagen (Figure 7E). In addition, when incubated on slides coated with Matrigel (analogous to the experiment depicted in Figure 7D), the cellular behavior again was identical to what had been observed in the other 2-D systems (unpublished data). These results suggest that it is indeed the dimensionality of the environment that critically influences the interaction of phagocytes with fungal pathogens.

Finally, it was important to investigate whether the observed phenomena were restricted to mouse phagocytes only. To analyze this, we investigated the interaction of human peripheral blood PMNs from three independent healthy donors with both fungal pathogens and in 2-D and 3-D environments. We found that the behavior of human cells closely mimicked the murine PMNs, in that phagocytosis of A. fumigatus was much better in the 2-D compared to the 3-D environment. We also noted that the interaction with C. albicans was exactly the opposite (Figure 7F). Thus, the dependence of phagocytes, at least of PMNs, on the dimensionality of the environment appears to be a general phenomenon not restricted to only mouse cells.

## Discussion

In this study we analyzed the cellular dynamics underlying the interaction and phagocytosis of two important fungal pathogens, A. fumigatus and *C. albicans,* using the major phagocytes of the mammalian body. Continuous imaging, which is indispensable for these studies, requires the reliable identification of both cells and fungi under constant light exposure. Expression of *DsRed2* under control of the *acuD*-promoter resulted in bright, stable fluorescence in conidia of *A. fumigatus,* which is necessary for long-term live-cell imaging analyses. At the same time, this transgenic fungus confirmed our earlier data on the environmental conditions leading to activity of the *acuD-*promoter [36], and thus adds additional proof to the efficacy of the fluorescent reporter gene strategy for studies on the activity of novel promoter elements in filamentous fungi [38]. The fact that the *acuD*-promoter was active during germination of conidia and outgrowth of macrophages made this promoter a valuable tool for following phagocytosis of conidia in cells.

The *acuD*p*-DsRed2* transgenic A. fumigatus made it possible to capture dynamic pictures of the phagocytosis process of conidia by the main cell types that interact with the fungus during natural infection [39]. This also made it possible to evaluate the fate of individual conidia and cells over a period of several hours and, finally, an assessment of phagocytic efficiency on a per cell basis could be conducted. The data indicate that the majority of cells were not able to phagocytose each conidium they touched. Although a number of cells, especially among AMs, were 100% successful (each contact led to phagocytosis of the touched conidium), a larger fraction of contacts between phagocytes and conidia did not end in phagocytosis, but rather in the release of conidia by the phagocytes. This could be observed not only with cells that had not phagocytosed any conidium, but also with cells that had already successfully phagocytosed conidia before. Hence, phagocytosis is not always the consequence of physical contact between a phagocyte and a pathogen, but could result from other, yet undisclosed, factors within the phagocyte and pathogen that need to coincide for phagocytosis to occur. This might explain why, in conventional phagocytosis assays of conidia, only ∼50% of AMs finally carry conidia [7], and after infection in vivo only 40%–50% of all airway DCs showed ingested conidia [17].

In addition, live microscopy also detected a novel, previously unrecognized means of phagocyte–pathogen interaction: the dragging of large numbers of conidial elements by individual cells without phagocytosing them. This was especially prominent among PMNs. The dragging of conidia by highly motile PMNs could lead to the collection of almost all conidia visible within a given field of view and to the formation of aggregates of conidia surrounded by highly motile PMNs. By this means, PMNs, which are not as efficient in phagocytosing conidia as AMs, might take control over large numbers of inhaled conidia. These aggregates have been isolated recently from infected murine lungs and shown to be associated with large amounts of reactive oxygen species [40], which could be a possible mechanism employed by PMNs to control *Aspergillus* infection [41]. Future work is needed to show whether such aggregates between PMNs and conidia can also be isolated from the lungs of humans exposed to *Aspergillus* conidia. The lack of PMN–*Aspergillus* aggregates in patients succumbing to the infection might explain why neutropenia is an especially dangerous condition for infection with A. fumigatus.

The most important finding of this study was the strong dependence of the phagocytic efficiency of PMNs and AMs (excluding DCs) on the dimensionality (i.e., 2-D or 3-D) of the environment, which was retained irrespective of the presence or absence of collagen. It is well known that in vivo DCs must take up conidia from airways and then migrate through the interstitial lung tissue to reach draining lymph nodes for antigen presentation to T cells [17,35,42]. Thus, DCs must be able to function in both environments. However, PMNs and AMs interact with inhaled conidia in the lung alveolus on the surface of the alveolar epithelium beneath a very thin, liquid SLL [30], a situation representing a typical 2-D environment. Nevertheless, the ability to phagocytose C. albicans shows that PMNs are not generally unable to function in 3-D environments. Strikingly, hyphae, the tissue invasive form of *A. fumigatus,* were also efficiently attacked by a large number of PMNs in our 3-D environment (unpublished data). In the light of these observations, it is tempting to speculate that phagocytes may have co-evolved with different pathogens or even with their morphotypes in a way to optimally recognize them in the environment where the encounter within the mammalian body is most likely to occur. The ability to differentiate between C. albicans morphotypes also was previously demonstrated for DCs [43], albeit only for 2-D environments. The inability of PMNs to recognize pathogens in the inappropriate environment may have important consequences. For example, mice, which can accept a lung infection with 2 × 10^8^ A. fumigatus conidia without notable dysfunction [44], are killed by 5 × 10^6^ conidia when administered intravenously [45]. Thus, mice are >40 times as sensitive to the same infection when the infection occurs in a non-natural environment despite the presence of a large number of PMNs in the bloodstream. This might also explain why the transition from a local to systemic infection, which occurs by hematogeneous spread of *A. fumigatus,* shows such high mortality [9].

Currently, the molecular explanation for these findings is puzzling. It has been shown that human macrophages can more efficiently phagocytose and kill bacteria [46] or *Candida* [13] in contact with a type-I collagen gel. This was associated with increased Fc-receptor or complement-receptor–mediated uptake and induction of phagolysosomal fusion after contact with collagen. However, the same effects obviously cannot account for the decrease in activity of PMNs against A. fumigatus in collagen. Generally, the cellular receptors responsible for ingestion of A. fumigatus by phagocytes are poorly defined. It has been shown that surfactant is important for this process [47]. However, since purified cells were used, participation of surfactant in the processes described here can be excluded. Previously it had been shown that PMNs were able to rapidly change their transcriptional profile in response to the environment [48]. Thus, the de novo expression, or down-regulation of receptors in 3-D compared to 2-D environments is likely. The same holds true for the pathogens themselves. For example, a simple temperature shift was shown to alter the expression of several hundred genes in A. fumigatus [49], so it is very likely that genes are differentially expressed in A. fumigatus after transfer of the culture from liquid culture into collagen. Such an adaptation might account for the inability of phagocytes to recognize the fungus in this environment. The ability to adapt the transcriptional profile to the environment was also shown in C. albicans [10,50]. Thus, future work needs to define genetic alterations in both the pathogen and the phagocyte in relation to the environment in order to obtain a complete understanding of the cellular processes underlying dragging, phagocytosis, and the killing of fungal pathogens in 2-D and 3-D environmental niches.

## Materials and Methods

### Fungal and bacterial strains.

CEA17 is a uracil-auxotrophic A. fumigatus strain, which encodes a mutated *pyrG* gene [51]. The A. fumigatus strain AcuDp-DsRed2 was derived from the CEA17 strain following transformation with plasmid pacuDp-DsRed2-pyrG. The strain carries two copies of the *acuD*p-*DsRed2* gene fusion integrated ectopically into the genome. Vectors and plasmids were propagated in Escherichia coli XL1 Blue MRF′ (Δ(*mcrA*)*183* Δ(*mcrCB-hsdSMR-mrr*)*173 endA1 supE44 thi-1 recA1 gyrA96 relA1 lac* [F′ *proAB lacI^q^ Z*Δ*M15* Tn10 (Tet^R^)]) (Fermentas, http://www.fermentas.com).

### Fungal cultivation conditions*.*

For the cultivation of A. fumigatus strains, AMM with 1% (w/v) glucose or 50 mmol ethanol as a carbon source was used [52]. Conidial suspensions were obtained from AMM agar plates after 5 d of cultivation [52]. Fungal and bacterial strains were grown at 37 °C. Spore suspensions were prepared as described without the addition of antibiotics [52]. E. coli strains were grown on LB agar plates or in LB medium at 37 °C. Ampicillin was added to give a final concentration of 100 μg/ml. C. albicans (based on SC5314, obtained from R. Calderone, Georgetown University) was grown to stationary phase in YPD medium (Sigma, http://www.sigmaaldrich.com/Brands/Sigma.html) at 30 °C with orbital shaking at 160 rpm. For fluorescence labeling, 1 × 10^8^ yeasts were harvested by centrifugation (16,000*g,* 5 min, 24 °C), washed twice in 1 ml PBS, and stained with CFSE (0.5 μmol in 1 ml PBS/0.1% DMSO) (Invitrogen, http://www.invitrogen.com) for 1.5 h at 37 °C. Yeast cells were washed three times in PBS to remove remaining dye before use.

### Generation of recombinant plasmids.

An A. fumigatus sequence containing the promoter from the *acuD* gene encoding isocitrate lyase [36] was used. The pDsRed2 vector was obtained from Clontech (http://www.clontech.com). The *A. fumigatus pyrG* gene was used as a selection marker [53]. The *pyrG* gene was synthesized by PCR amplification using the oligonucleotides pyrG_Afum_notI_1 (5′-GCGGCCGCACAGCTATGCGACCG-3′) and pyrG_Afum_notI_2 (5′-GCGGCCGCATATCTCTGGTTGGAG-3′), which encode NotI restriction sites (underlined) and chromosomal DNA of A. fumigatus as the template. For generation of the *acuD*p*-DsRed2* gene fusion the 5′ sequence of the A. fumigatus isocitrate lyase gene (942 bp), including the ATG, was amplified by PCR, using oligonucleotides AfAcuD_upst_Bam (5′-CGGATCCGAAGGACAGGAAC-3′) and AcuD_rev_KpnI (5′-CTGGATCCAAACC*CAT*TGTGACAGGTATGAAGAGG-3′) and chromosomal DNA of A. fumigatus as the template. The oligonucleotides encoded BamHI and KpnI sites at the 5′ and 3′ ends, respectively (underlined). The PCR fragment was cloned into the pCR2.1 vector. The resulting plasmid was digested with BamHI and KpnI. The DNA fragment obtained was ligated into plasmid pDsRed2, which had also been digested with BamHI and KpnI, to give the plasmid pacuDp-DsRed2. The PCR amplified *pyrG* gene was integrated into plasmid pacuDp-DsRed2 by ligation into its single NotI site to yield plasmid pacuDp-DsRed2-pyrG (Figure 1B). Transformation of A. fumigatus was performed as described [54].

### Fluorescence and light microscopy.

Microscopic analyses shown in Figure 1 were performed with a Leica DM4500 B microscope with a filter set of BP 546/12 for excitation and BP 605/75 for emission. Images were obtained and processed with Leica Application Suite 2.3.4 R2 (Leica Microsystems, http://www.leica-microsystems.com). Fluorescence analysis of Figure 1G was performed with an Axiovert 200 M/LSM 510 META laser scanning confocal microscope (Zeiss, http://www.smt.zeiss.com). DsRed2 and 4′,6-diamidino-2-phenylindole-dihydrochloride (DAPI) were excited by a laser line of 542 and 364 nm, respectively. Fluorescence signals were detected using a 385-nm long pass filter for DAPI and by 560–615-nm band pass filters for DsRed2. Images were acquired using the LSM-510-META 3.2 software (Zeiss). Figures were assembled with Adobe Photoshop (http://www.adobe.com).

### Confocal microscopy.

PMNs and DsRed A. fumigatus conidia were mixed at a ratio of 1:5 and incubated for 1 h over 12-mm poly-L-lysine–coated cover slips. Cells were fixed in 4% paraformaldehyde (PFA) (Sigma) at (pH 7.4) for 20 min at room temperature and then washed three times with prewarmed PBS. The cells were then permeabilized with 4% PFA and 0.1 % Triton X-100 (Sigma). After washing with PBS, the cells were blocked with a solution of PBS containing 1% BSA and 5% horse serum (Sigma). Staining for actin cytoskeleton was done using Alexa 488 labeled phalloidin (2 U/ml) (Molecular Probes, http://probes.invitrogen.com) for 45 min to 1 h. Cover slips were mounted on clean glass slides with Mowiol (Calbiochem, http://www.emdbiosciences.com) with 0.01% paraphenylene diamine (Sigma). Cells were imaged with an Olympus LSM confocal microscope (Fluoview 1000) with a 100-× objective. A 3-D rendering of confocal z stacks was done using the Volocity software package (version 4.0; Improvision, http://www.improvision.com).

### Cell preparation.

BALB/c bone marrow DCs were generated in 8-d cultures as described [55]. Cell lines secreting murine granulocyte-macrophage colony-stimulating factor (GMCSF) or IL-4 were kindly provided by Thomas Blankenstein from the Max Delbrück Center for Molecular Medicine (MDC), Berlin (http://www.mdc-berlin.de). PMNs were obtained by positive selection from mouse bone marrow (BM). BM cells were prepared by flushing the femurs and tibiae of BALB/c mice with PBS + 1% FCS (v/v). Following erythrocyte lysis, the cells were incubated with Fc–Block (BD Biosciences, http://www.bdbiosciences.com) and then subjected to cell sorting by Gr-1–labeled magnetic particles (clone RB6-8C5, BD Biosciences) following the manufacturer's instructions. The purity of the cells was >97% as determined by FACS analysis. AMs were obtained by washing the trachea and lungs of BALB/c mice with PBS through a 22G plastic catheter (Braun, http://www.bbraun.de) to obtain bronchoalveolar lavage fluid. After erythrocyte lysis, the cells were resuspended in complete medium supplemented with glutamine, penicillin, and streptomycin. The cells were kept on ice until use.

J774 cells were cultured in BioWhittaker's X-Vivo 15 medium (Cambrex, http://www.cambrex.com). Before use, the cells were stimulated overnight with 2.5 U/ml interferon-γ (Boehringer Ingelheim, http://www.boehringer-ingelheim.com). RAW 264.7 macrophages [37] (American Type Culture Collection, http://www.atcc.org) were maintained in RPMI (Gibco, http://www.invitrogen.com) containing 10% FCS at 37 °C and harvested by scraping with a rubber policeman. The cells were subjected to no more than 20 passages.

Human PMNs were derived from the peripheral blood of healthy volunteers. Briefly, freshly drawn blood was diluted with HBSS without CaCl_2_ and MgCl_2_ (Gibco) and layered over PolymorphPrep (Axis-Shield PoC AS, http://www.axis-shield.com) according to the manufacturer's instructions. PMNs were carefully removed and resuspended in RPMI supplemented with 5% pooled human serum (Chemicon, http://www.chemicon.com) following washing and erythrocyte lysis with ACK buffer (Cambrex). All experiments with human cells were done with 5% (v/v) pooled human serum.

For outgrowth experiments of germinating conidia from macrophages (Figure 1G), J774 macrophages were cultured in RPMI complete medium (Cambrex) + 5% (v/v) FCS (= RPMIF). Macrophages were incubated with a ratio of two conidia per macrophage for 2 h. The cells were washed extensively with RPMIF and incubated for 4 h with RPMIF containing 25 mmol imidazole. The cells were then fixed with 3.8% (v/v) para-formaldehyde for 10 min at room temperature and stained with DAPI. The samples were analyzed with fluorescence microscopy.

### Time-lapse video microscopy.

A total of 1 × 10^6^ purified cells were mixed with 0.5–1 × 10^7^ filtered (BD Falcon cell strainer) conidia of strain AcuDp-DsRed2 or 3 × 10^6^ *Candida* yeasts in 66 μl complete medium (CM) containing RPMI 1640 supplemented with NEAA (1 ×), FCS (10%, v/v), L-glutamine (2 mmol), HEPES (10 mmol), sodium pyruvate (1 mmol), β-mercaptoethanol (50 μmol), and penicillin/streptomycin (100 U/ml). The suspension was mixed with 133 μl of type-I collagen stock solution (Vitrogen-100; Nutacon, http://www.nutacon.nl) to a final collagen concentration of 1.7 mg/ml and poured into a tracking chamber as described [23,24]. Fluorescence and cell interactions were monitored simultaneously at 37 °C at two frames/min using an Olympus BX61 microscope with a 60 × LUMPLFL W/IR (NA 0,9) lens, together with the cellR software (version 2.1) from Olympus Biosystems (http://www.olympus-europe.com). For observations in a liquid medium, immune cells and conidia were mixed at a ratio as mentioned above to a final volume of 200 μl in complete medium. These cell suspensions were poured into glass chambers, and microscopy was performed focusing on the bottom of the chamber. Alternately, imaging of cells and pathogens was carried out over collagen-coated surfaces that were prepared by applying a very thin coat of type-I collagen at a concentration of 1.7 mg/ml on a glass slide to rule out the effect of collagen on 2-D systems. Matrigel basement membrane matrix (BD Biosciences), containing laminin as a major component, was used as an alternative for the type-I collagen matrix and was diluted with a mixture of cells and respective pathogens in PBS with 1% BSA at the ratio as mentioned above, so that its final concentration was 1.7 mg/ml.

### Analysis of cell migration, phagocytosis, and dragging efficiency.

Cell migration was analyzed by computer-assisted cell tracking using a software program developed for this study as described [21,56]. Briefly, the time-lapse video was displayed on a computer screen. For videos in a collagen matrix, this was a 2-D projection of a 3-D image. From 40 to 60 cells were then randomly marked to give an unbiased representation of the cell population. The cell movements were then followed with a trackball in both media and collagen matrices. Tracking was stopped in case a cell disappeared within the collagen matrix or left the field of view. From these tracks cell velocities were calculated and normalized to the true dimensions of the field of view and expressed as micrometers per minute. In addition, the percentage of cells migrating at a given time point were expressed as activity. The efficiency of phagocytosis and dragging was obtained as follows: all cells visible in the video sequences were analyzed for their physical association with conidia (touching). Then it was further observed whether this touch led to phagocytosis of the conidium, which could last from a few seconds up to several hours. The ratio of completed phagocytosis events over the number of observed touches was calculated as PTI for each cell. The DTI was calculated in a similar manner for interactions that did not lead to phagocytosis but nevertheless showed considerable displacement of conidia, often over hundreds of micrometers.

### Electron microscopy.

Samples were fixed in 5% formaldehyde and 2% glutaraldehyde in cacodylate buffer (0.1 M cacodylate, 0.01 M CaCl_2_, 0.01 M MgCl_2_, and 0.09 M sucrose [pH 6.9]) for 1 h on ice and washed with cacodylate buffer. We coated 12-mm cover slips with poly-L-lysine (Sigma) for 10 min, and then they were washed in distilled water and air dried. We placed 30 μl of the fixed samples on a cover slip and allowed it to settle down for 10 min. Cover slips were then fixed in 2% glutaraldehyde in cacodylate buffer (5 min) and washed with TE-buffer (20 mM TRIS and 1 mM EDTA, [pH 6.9]) before dehydrating in a graded series of acetone (10%, 30%, 50%, 70%, 90%, and 100%) on ice for 15 min for each step, critical-point dried with liquid CO_2_ (CPD 30; Balzers, http://www.oerlikon.com) and covered with a gold film by sputter coating (SCD 40, Balzers), before being examined in a field emission scanning electron microscope (Zeiss DSM 982 Gemini) using the Everhart Thornley SE detector and the inlens detector in a 50:50 ratio at an acceleration voltage of 5 kV.

### Statistics.

The values for cells phagocytosing conidia in a collagen culture versus in a liquid culture were analyzed for significant differences using the nonparametric Mann-Whitney *U*-test. Several data contained only zero values, which precluded the use of the Mann-Whitney *U*-test. In such cases, the Wilcoxon rank sum test was used to estimate significance. All other statistical analyses were done using the Student's unpaired *t*-test. *p*-Values < 0.05 were considered significant (*), values < 0.01 highly significant (**).

## Supporting Information

Video S1 Polymorphonuclear Cells with Conidia in Liquid Media A rapidly moving neutrophil can be seen taking up several conidia over an imaging time of 2 h with one frame every 30 s.
(2.5 MB AVI)

Video S2 Dendritic Cells with Conidia in Media A single DC can be seen here interacting with conidia in liquid media and taking up two of them. The observation was made over a period of 3 h with a time lapse of 30 s.
(6.2 MB AVI)

Video S3 Alveolar Macrophages with Conidia in Liquid Medium Two highly active alveolar macrophages can be seen ingesting conidia. Time lapse is 30 s per frame over 2.5 h.
(4.1 MB AVI)

Video S4 J774 Cells with Conidia in Liquid Media An active J774 macrophage is seen taking up at least three conidia in a cooperative manner. The J774 cells were treated with 5 ng/ml interferon-γ one night before filming with conidia. The observation was made over a period of 2.5 h every 30 s.
(2.9 MB AVI)

Video S5 PMNs with Conidia in Collagen A PMN can be seen here touching a conidium and even displacing it, but eventually moving on without further interaction. Imaging time was 3 h with one frame every 30 s.
(671 KB AVI)

Video S6 Dendritic Cells with Conidia in Collagen A single DC can be seen here efficiently taking up at least four conidia in its vicinity.
(5.4 MB AVI)

Video S7 PMNs Dragging Conidia in Media Several neutrophils can be seen dragging clusters of conidia without actually phagocytosing them. A majority of conidia are attached to PMNs at the end of the video leading to the formation of an aggregate. Imaging time was 3 h with a time lapse of one frame every 30 s.
(3.1 MB AVI)

Video S8 Dendritic Cells Dragging Conidia in Collagen A well resolved dendritic cell drags a conidium through a distance of up to 9 μm. The conidium, however, is not phagocytosed by the cell. The observation was made over 3 h with one frame every 30 s.
(14.0 MB AVI)

Video S9 Conidia Can Be Attached to the PMN Surface or Truly Internalized A 3-D reconstruction of a z stack showing a fixed preparation of a PMN carrying five conidia intracellularly and one on the surface. The actin cytoskeleton of the PMN is seen here in green, while the conidia are seen in red.
(10.5 MB AVI)

Video S10 PMNs with C. albicans Yeasts in Liquid Media Neutrophils are not able to take up C. albicans yeasts despite several contacts in liquid media, in contrast to A. fumigatus conidia. In addition, no dragging of yeasts is visible here.
(2.0 MB AVI)

Video S11 RAW Macrophages with Yeasts in Media Like neutrophils, RAW macrophages as seen here are unable to take up yeasts despite contacts. Imaging time was 3 h with an interval of 30 s after each frame.
(2.9 MB AVI)

Video S12 PMNs with C. albicans Yeasts in Collagen Neutrophils are seen here interacting with C. albicans in collagen matrix. In contrast to A. fumigatus conidia, most of the PMNs carry *Candida,* and a phagocytosis event can be seen at 142.5 min.
(6.9 MB AVI)

Video S13 RAW Macrophages with Yeasts in Collagen A few RAW macrophages can be seen here carrying *Candida* yeasts. A large yeast is taken up by a cell at 30 min and is pulled up towards the cell. The observation was made over a period of 2 h with one frame every 30 s.
(1.6 MB AVI)

Video S14 Competitive Phagocytosis Assay in Media A neutrophil is seen here with a heavy load of A. fumigatus conidia, which are dragged along with the cell. The PMN seems to selectively pick up conidia despite several contacts with *Candida* yeasts. The video was made over 2 h with a time lapse of one frame every 30 s.
(6.9 MB AVI)

Video S15 Competitive Phagocytosis Assay in Collagen A neutrophil can be seen here selectively taking up *Candida* yeasts despite several contacts with A. fumigatus conidia in a 3-D collagen matrix. Imaging time was 2 h with an interval of 30 s after every frame.
(2.6 MB AVI)

### Accession Numbers

The National Center for Biotechnology Information (NCBI) CoreNucleotide (http://www.ncbi.nlm.nih.gov) accession numbers for the genes and gene products discussed in this paper are *acuD* (gi|44844013) and *acuD* promoter (AJ620297).

## Abbreviations

2-D: two-dimensional
3-D: three-dimensional
AM: alveolar macrophage
AMM: *Aspergillus* minimal medium
CFSE: carboxyfluorescein-succinimidyl-ester
DAPI: 4′,6-diamidino-2-phenylindole-dihydrochloride
DC: dendritic cell
DTI: dragging touching index
PMN: polymorphonuclear neutrophil
PTI: phagocytosis touching index
SD: standard deviation
SLL: surface-lining layer
